# In Nature's School

**Published:** 1889-06-22

**Authors:** 


### The Doctor's Pulpit.

### IN NATURE'S SCHOOL.

If it be true that "all the world's a stage, and all the men and women merely players," it is true in a still deeper sense that all the world's a school, and all the men and women and children are merely pupils. Very dull pupils many of us are, too—so dull that not a few have even failed to recognise that the world is a school at all. To some it is a mere toilsome workshop, with a master who exacts a certain return of muscle power for his Saturday's wages. To others it is a stiff fallow, which they must plough lengthwise and crosswise, and harrow and sow with huge toil, though they know that almost all the profit of their labour will go into other pockets than their own. An increasing number of our fellow-beings look upon the world as a vast playroom, in which he who secures the largest quantity and the most piquant quality of amusement or pleasure is the most successful. This latter class consists much more largely of women than of men. To do them justice most men, even of the idle classes, have the grace to be ashamed of a life of unmitigated idleness, and will seek some kind of more or less useful occupation in order that they may at last delude their consciences with the idea that they are at work. But women, in unlimited numbers, seem to find no difficulty whatever in making the whole of life one long incessant and unredeemed "frivvle."

The prevailing natural characteristics of boys and girls in elementary schools are two—idleness and mischief. Whether man be descended from the ape or not, there is no doubt that at his immature stage, which we may suppose corresponds more or less to the mental condition of the adult monkey, he has a fine capacity for fun. Amusement is not only the breath of his nostrils, but the occupation of his thoughts and the work of his hands. Nature is sometimes a stern schoolmistress, but she does not often visit the young with smart strokes of punishment. She provides them rather, not only with the faculty of being readily amused, but with all kinds of objects on which that faculty may exercise itself. The kitten's tail, for example, is a temptation to the two-year-old infant which he can by no means resist. Later on the blackbird's nest in the hedge or the woodpigeon's in the forest tree offer eagerly relished opportunities for the development of the sporting instinct. Even at the age of early manhood Nature is still indulgent, and in countries not denaturised by civilisation, she makes food and warmth often depend on the diligent use of the natural faculty possessed by man for hunting and trapping the wild creatures of the forest and the sea.

On entering a new school, the intelligent boy or girl soon understands two things—the temper and character of the teacher, and the opportunities the school offers for practical advancement. The school of Nature is, however, a very large institution, and Mistress Nature herself is not quite so easily comprehended in all her various moods as a village dominie. But it is very necessary to comprehend her, for the only obedience she will be satisfied with is that of the intelligent kind. Stupid fellows, especially those who have come to maturity and self-dependence, fare very badly at her hands. The stupid and the idle are her pet aversions. The self-willed, too, she is very stern with. A good many human creatures have such a wondrous idea of their own powers and merits that they would think nothing of making the sun stand still in the heavens every day and all day long for their special convenience. But Mistress Nature will not hear of any deviations from her ancient habits to please either cabinet ministers, doctors, or patients. Not even for lawyers and judges, who it is well known hold the keys of all authority in their hands, will she step a single foot aside from her accustomed path. This peculiarity of hers, whereby she invariably insists upon having her own way, must be both understood and reckoned with if a man wishes to be either prosperous or well. There are some foolish creatures who never will understand it. They have made up their minds that they are very meritorious, very great, and very distinguished persons, and they insist in their own minds upon being taken at their own valuation. Sometimes these persons are kings; sometimes only corporals of dragoons. They do not care for Nature—not they. If it be hinted that their intellect is not very great, that their merits are not conspicuous, and that their doings have never been such as to compel the attention and admiration of their fellow-men, the only reply such a hint receives is a look of contemptuous indignation. A mere man may feel himself annihilated by such a look, but Mistress Nature does not even smile. She seems to say, "If the man will hold his head so high that he cannot see the water into which he is walking he must even drown." Reason, by seeking to understand and to obey the dictates of Nature, conquers her in the end and makes her a willing servant and friend stupidity and obstinacy, though they may enter into most desperate conspiracies, can never fight anything but a losing battle with her.

The best primary or finishing school is the school of Nature: the noblest university is the University of the World. Whole generations of schoolmasters and learned professors have rebelled against her, and set up standards of their own, again and again. But she has always compelled succeeding generations to return to their allegiance; and they have seen and laughed at the folly of their predecessors. This grand school is a kind of kindergarten on an enormous scale. The system of teaching is by object-lessons, and the various lessons constitute an ordered series of things. Mere words and theories are of no importance, except as means to the explaining and understanding of things. Nature never makes the mistake of regarding a word as equal to a thing, of considering grammar as important as the grammarian, or even as the grammarian's digestion. The mathematician, or the zoologist, or the student of philosophy may be so foolish as to think the world runs or ought to run on his particular lines, and that if it did so run all would be well. "You are but foolish children," says Nature. "The world is fashioned to run on my lines, and cannot be made to run on any other." The wisdom of men and women is to put themselves in a line with Nature; to make an offensive and defensive treaty with her. When that is done, it is wonderful how docile and helpful she is. She will furnish wind for the mill, or for the driving of ships across the sea. She will produce corn, and wine, and oil in abundance. She will restore the sick to health, and make the healthy stronger and better than they were before. But trifled with she will not be. Let any man, or any ten thousand men, resolve to fly in her face, and she can drown them in a storm, dash them to pieces on the rocks, kill them with cholera or fever, or decimate them in a hundred ways.

The world, we say, is an ordered system, a system made to move in regulated succession, a system that contains within itself the power of resistless and continuous movement. It must and will go on, like the mill that is made to grind corn, and grinds it if it once gets between the stones, whether the corn likes it or not. No plea of necessity or merit can prevent the reduction of the corn to powder when once it is well within the iron grasp of the rollers. In the matter of bodily health and well-being, Nature seems almost more determined to have her own way than in any other business. Rebellion against her reasonable canons is common, but punishment is quite as common. There are men who, like successful swindlers, avoid the law for a long time; but, like swindlers also, they receive the severer punishment when the inevitable day of reckoning finally dawns. Some people are foolish enough to think this is mere talk. The doctor has so many columns to fill and must fill them with something. Every man of reason and insight knows better. Whoever trifles, Nature does not; whoever halts, she moves on; whoever opposes her, she tramples down. The hard facts of the world—the summer, the wintei, the darkness, the daylight, the storm, and the midday heat, the earthquake and the pestilence, the mountains and the valleys, the wild beasts and the cattle, the trees and the grass, love and hatred, birth and death—these and their like constitute the world in which man must live, and in which he must live in a well-defined way with due regard to all that lives and works around him.

City life blinds men to the facts of Nature, and thus diminishes their intelligence. It substitutes a kind of sharpness and cunning in small things for the broader common-sense and experience that takes in all the facts that influence men's lives. The city man tends to become like the street arab, preternaturally quick at trifles; but he is often as little fitted to cope with serious difficulties—difficulties that demand patient intelligence and reasoning—as the street arab is to meet the ploughboy in a game of fisticuffs. In a stand-up fight the gamin goes down before the countryman like a pack of cards; and similarly the town man, who has confined his thoughts strictly to his little specialty, is lost and bewildered when a great human crisis comes upon him. The broad school of Nature trains men better than that.

